# Comparative analysis of bile metabolic profile in patients with biliary obstruction complicated by *Clonorchis sinensis* infection

**DOI:** 10.3389/fcimb.2023.1254016

**Published:** 2023-09-12

**Authors:** Xueli Zhang, Su Han, Xu Jiang, Shanshan Duan, Yannan Gao, Jian Ding, Xiang Li, Beibei Sun, Xinyi Hu, Xiaoli Zhang, Weizhe Zhang

**Affiliations:** ^1^ Department of Parasitology, Harbin Medical University, Harbin, China; ^2^ Department of Public Health and Preventive Medicine, Wuxi School of Medicine, Jiangnan University, Wuxi, China; ^3^ Beijing Obstetrics and Gynecology Hospital Capital Medical University, Beijing Maternal and Child Health Care Hospital, Beijing, China; ^4^ Department of Orthopaedic Surgery, The Fourth Affiliated Hospital of Harbin Medical University, Harbin, China; ^5^ Clinical Laboratory, Zhuhai Maternal and Child Health Hospital, Zhuhai, China; ^6^ Department of Stomatology, Laixi People's Hospital, Shandong, China

**Keywords:** biliary obstruction, *Clonorchis sinensis*, bile, non-targeted metabolomics, targeted metabolomics

## Abstract

**Background:**

Clonorchiasis is an important foodborne parasitic disease. However, eggs of *Clonorchis sinensis (C. sinensis)* cannot be detected in feces during biliary obstruction. Moreover, many diseases can cause biliary obstruction, such as gallstones, adenocarcinoma, cholangiocarcinoma and *Ascaris lumbricoides* infection. Therefore, it is of great significance to distinguish between patients of biliary obstruction and biliary obstruction with *C. sinensis* infection.

**Methods:**

A total of 48 biliary obstruction patients were enrolled, including 23 infected with *C. sinensis (C. sinensis)* (OB+C.s) and 25 non-infected subjects (OB). The bile samples were collected by endoscopic retrograde cholangiopancreatography and analyzed using ultra-high-performance liquid chromatography-quadrupole time-of-flight mass spectrometry (UHPLC-QTOF MS). Additionally, multivariate statistical analysis methods were employed to identify differential metabolites. Next, bile amino acid levels were determined by targeted metabolomics analysis.

**Result:**

A total of 146 and 132 significant metabolites were identified in electrospray ionization (ESI)+ and ESI− modes, respectively. The levels of amino acids (asparagine, glutamate, ornithine) and polyamines (spermidine and spermine) were significantly changed. Targeted analysis showed that the levels of amino acids (such as L-arginine, L-glutamine, L-lysine, L-propionic, and L-tyrosine) were lower in OB+C.s patients compared to those in OB patients. Marked metabolic pathways were involved in “Glutathione metabolism”, “Caffeine metabolism”, “Alanine, aspartate and glutamate metabolism”, “Arginine and proline metabolism”, “Purine metabolism”, “Beta-Alanine metabolism”, and “D-glutamine and D-glutamate metabolism”.

**Conclusion:**

These results show that there were significant differences between OB+C.s and OB patients, especially in amino acids. The metabolic signature and perturbations in metabolic pathways may help to better distinguish OB+C.s and OB patients.

## Introduction

*Clonorchis sinensis*, a vital foodborne parasitic, causes clonorchiasis endemic in Asian countries and infected approximately 35 million people (Lun et al., 2005; Lim et al., 2006). Humans are the final host and become infected by ingesting raw freshwater fish containing metacercaria (Lun et al., 2005). Metacercariae of *C. sinensis* excyst in the duodenum of mammalian hosts, move to the intrahepatic bile duct, and develop into adults in the bile environment (Qian et al., 2016). Heavy and chronic clonorchiasis often results in the loss of liver function, chronic liver diseases, and even cholangiocarcinoma (CCA) (Hong et al., 2003; Rim, 2005). The gold standard for diagnosing *C. sinensis* infection is finding eggs in stool (Qian et al., 2016). However, eggs cannot be detected in feces during biliary obstruction (Attwood and Chou, 1978; Sripa et al., 2010). Moreover, many diseases can cause biliary obstruction, such as gallstones, pancreatic adenocarcinoma, CCA, and *Ascaris lumbricoides* infection. Therefore, it is of great significance to distinguish between patients of biliary obstruction (OB) and biliary obstruction with *C. sinensis* infection (OB+C.s). Adult *C. sinensis* parasitize in mammals’ bile ducts, where they are exposed to bile all their entire lives (Dai et al., 2020). We assumed that adult *C. sinensis* continuously interact with bile components.

Bile, synthesized in the liver and stored in the gallbladder, is mainly constituted by bile acids, cholesterol, phospholipids, and proteins. Numerous endo-/exogenous metabolites and bioactive compounds are delivered to the gut via bile (e.g., bile acids and phospholipids) in enterohepatic circulation (Karlsen, 2016). Previous studies have shown that metabolomics analysis made great progress in CCA (Sharif et al., 2010), primary sclerosing cholangitis (Tietz-Bogert et al., 2018), and biliary strictures (Urman et al., 2020), difference metabolites were significantly transformed in patients compared with the benign disease groups. Alsaleh et al. showed that the serum metabolism in CCA exhibited changes in metabolites related to inflammation, altered energy production, and phospholipid metabolism (Alsaleh et al., 2020). However, no study reported the difference of metabolites in bile between OB and OB+C.s patients.

Endogenous small-molecule metabolites (<1 kDa) are the end products of interactions between genes and the environment (Kori et al., 2016), reflecting the metabolic response to pathophysiological stimuli or even genetic alteration at a certain time point (Chang et al., 2015). Small-molecule metabolites in biological systems are measured using metabolomics, which provides crucial information on the dynamic metabolic reactions of endogenous components during the progression of disease (Chang et al., 2015; Guo et al., 2018). There are two primarily analytical methods in conducting metabolomics studies: untargeted and targeted (Dettmer et al., 2007; Patti et al., 2012). Untargeted metabolomics typically investigates a vast number of metabolites in a sample without bias and can be used to identify unknown metabolites, whereas targeted metabolomics frequently concentrates on particular known metabolic pathways and can be used to quantify metabolites (Dudley et al., 2010; Patti et al., 2012). To obtain a highly informative profiling of the entire metabolome is difficult due to the chemical and physical diversity of metabolites (Torres et al., 2017). Therefore, integrative adoption of two or more approaches is required for a more comprehensive and accurate metabolic profile. For these reasons, we combine the untargeted and targeted mass spectrometry (MS)-based metabolomics methods to analyze the metabolic profile of bile in OB and OB+C.s patients.

In our study, we first utilized an untargeted ultra-high-performance liquid chromatography-quadrupole time-of-flight mass spectrometry (UHPLC-QTOF MS) approach to perform metabolomics analyses between OB and OB+C.s patients. Then, to further measure amino acid metabolism, a targeted ultra-high-performance liquid chromatography multiple reaction monitoring tandem mass spectrometry (UHPLC-MRM-MS/MS) method was adopted. The aim of this study was to assess the bile metabolic profile from patients of OB and patients of OB+C.s using untargeted and targeted MS-based metabolomics approaches. Our results preliminarily revealed the differences of bile metabolism between patients of OB and OB+C.s. Meanwhile, this present study supplied new insights into the molecular mechanisms of host–parasite interactions.

## Materials and methods

### Ethics statement

All protocols and procedures of this study conformed to the ethical guidelines outlined in the 1975 Declaration of Helsinki as reflected *a priori*. Prior to inclusion in this study, all participants provided written informed consent. The ethics committee of Harbin Medical University approved this study. All experiments were carried out in accordance with the approved guidelines and regulations.

### Subject enrollment and sample collection

In this study, 48 patients (24 men and 24 women, ranged from 32 to 79 years) with biliary obstruction diseases were enrolled from the Second Affiliated Hospital of Harbin Medical University. Whether caused by *C. sinensis* infection or not, all biliary obstruction patients had undergone endoscopic biliary drainage. The exclusion criteria included malignant bile duct obstruction due to acute suppurative cholangitis, pancreatic cancer, and CCA; chronic hepatitis or liver disease with functional damage; known active infections such as viral, bacterial, or fungal infections; other diseases such as any kind of tumor or uncontrolled chronic diseases involving the liver, heart, lung, and kidney.

The patients’ bile samples were obtained during endoscopic retrograde cholangiopancreatography (ERCP). All of the biliary drainage procedures were carried out by one physician. The bile samples were centrifugated at 10,000g for 10 min at 4°C, and the supernatant was immediately stored at -80°C until analysis.

### Diagnosis of infection with *C. sinensis*

The detection of *C. sinensis* eggs in the microscopic inspection of the bile pellet could establish the infection. Firstly, 1,000-μL bile sample was centrifuged in Eppendorf (EP) tube at 12,000 r/min for 10 min, the supernatant was discarded, and the bile sediment was resuspended with 100-μL Phosphate Buffer Solution (PBS). Next, 20-μL suspension was smeared, and three smears were done for each specimen. **Supplementary Figure S1** shows the *C. sinensis* eggs detected in the bile sample of OB+C.s patients.

### Bile inflammatory cytokine analysis

Commercial ELISA kits (eBioscience, San Diego, CA, USA) were used to measure the levels of inflammatory cytokines (interleukin-6 (IL-6), IL-10, tumor necrosis factor-Alpha (TNF-α) and Transforming growth factor- Beta (TGF-β)) in bile according to the manufacturer’s recommendations. The inflammatory cytokines of bile in the two groups were statistically analyzed using GraphPad Prism software.

### Preparation of bile sample and metabolite extraction

Two hundred microliters of bile samples was accurately measured out in an Ep tube, then 800-μL extract solution (acetonitrile:methanol = 1:1) including isotopically labeled internal standard mixture was added. After the mixture was vortexed for 30 s, it was sonicated for 30 min (5°C, 40 kHz). The mixture was allowed to settle at -20°C for 30 min. After centrifugation at 13,000g at 4°C for 15 min, the supernatant was removed and dried it with nitrogen. The mixture was redissolved using a 100-µL acetonitrile:water (1:1, v/v) solution, then the mixture was vortexed for 30 s and sonicated for 5 min (5°C, 40 kHz). After centrifugation at 13,000g at 4°C for 10 min, the supernatant were transferred to sample vials for LC-MS/MS analysis. The quality control (QC) samples were collected by mixing an equal aliquot of the supernatants from all samples.

### Untargeted serum metabolomics analysis

The metabolites were separated by chromatography using a Vanquish Horizon system (Thermo Fisher Scientific, USA) with an ACQUITY UPLC HSS T3 column (100 mm × 2.1 mm i.d., 1.8 µm; Waters, Milford, USA). The mobile phases consisted of 0.1% formic acid in water with formic acid (0.1%) (solvent I) and 0.1% formic acid in acetonitrile:isopropanol (1:1, v/v) (solvent II).

The solvent gradient changed according to the following conditions: from 0 to 3.5 min, 100% (I) to 75.5% (I): 24.4% (II); from 3.5 to 5 min, 75.5% (I): 24.4% (II) to 35% (I): 65% (II); from 5 to 5.5 min, 35% (I): 65% (II) to 100% (II); from 5.5 to 7.6 min, 100% (II) to 48.5% (I): 51.5% (II); from 7.6 to 7.8 min, 48.5% (I): 51.5% (II) to 100% (I); from 7.8 to 10 min, 100% (I) to 100% (I) for equilibrating the systems. The sample injection volume was 2 µL, and the flow rate changed according to the following conditions: from 0 to 7.4 min, the flow rate was set to 0.4 mL/min; from 7.4 to 7.8 min, the flow rate was set to 0.6 mL/min; from 7.8 to 9.0 min, the flow rate was set to 0.5 mL/min; from 9.0 to 10.0 min, the flow rate was set to 0.4 mL/min. The column temperature was maintained at 40°C. All samples were stored at 4°C during the period of analysis.

Thermo UHPLC-Q Exactive Mass Spectrometer with an electrospray ionization (ESI) source operating in either positive or negative ion mode was used to acquire the mass spectrometric data. The optimal conditions were set as follows: aus gas heater temperature, 425°C; aus gas capillary temperature, 325°C; sheath gas flow rate, 50 psi; aus gas flow rate, 13 psi; ion-spray voltage floating (ISVF), -3,500 V in negative mode and 3,500 V in positive mode; normalized collision energy, 20-40-60 V rolling for MS/MS. The Data Dependent Acquisition (DDA) mode was used for data collecting. The mass range covered by the detection was 70–1,050 m/z.

### Data preprocessing and annotation

The raw data were imported into Progenesis QI 2.3 for peak detection and alignment following UPLC-TOF/MS analysis. The peak intensity, mass-to-charge ratio (m/z), and retention time (RT) values were all produced as a result of the preprocessing. Metabolic features observed at least 80% of the time in any set of samples were retained. Following filtering, minimum metabolite values were imputed for particular samples in which the metabolite levels fell below the lower limit of quantitation and each metabolic feature was normalized by total. For data QC, the internal standard was employed, and metabolic features with a relative standard deviation (RSD) of QC >30% were ignored. Following normalization procedures and imputation, statistical analysis was performed on log-transformed data to find significant differences in metabolite levels between the two groups. These metabolic features’ mass spectra were found by using the accurate mass, MS/MS fragment spectra, and isotope ratio differences with scanning trustworthy biochemical databases like the Human Metabolome Database (HMDB) (http://www.hmdb.ca/) and Metlin database (https://metlin.scripps.edu/). Concretely, the mass tolerance between the measured m/z values and the exact mass of the components of interest was ±10 ppm. Only the MS/MS fragments scoring above 30 for metabolites with MS/MS confirmation were regarded as confidently identified. Otherwise, the metabolites were merely speculative.

### Multivariate statistical analysis

On the Majorbio Cloud Platform (https://cloud.majorbio.com), multivariate statistical analysis was carried out utilizing the ropls (Version1.6.2, http://bioconductor.org/packages/release/bioc/html/ropls.html) R package from Bioconductor. An unsupervised method, principal component analysis (PCA), was used to obtain an overview of the metabolic data, general clumping, trends, or outliers visualized. All of the metabolite variables were scaled to unit-variances prior to conducting the PCA. To compare the two groups’ global metabolic changes, orthogonal partial least squares discriminant analysis (OPLS-DA) was employed for statistical analysis. Prior to running the OPLS-DA, all of the metabolite variables were scaled to pareto scaling. Model parameters R2 and Q2, which offer data on the model’s interpretability and predictability, respectively, and mitigate the risk of overfitting, were used to assess the model’s validity. Variable importance in the projection (VIP) was calculated in the OPLS-DA model. *P* values were estimated with paired Student’s t-test on single dimensional statistical analysis.

### Differential metabolite analysis

Statistically significant between the two groups were selected with VIP value >1 and *P* value <0.05. Through metabolic enrichment and route analysis based on database searches (Kyoto Encyclopedia of Genes and Genomes, http://www.genome.jp/kegg/), the differential metabolites between the two groups were compiled and mapped into their respective biochemical pathways. These metabolites can be categorized based on the pathways they involved or the functions they carried out. Typically, enrichment analysis was used to determine a group of metabolites in a metabolism pathway whether it appears or not. The principle was that an annotated analysis of a single metabolite would eventually lead to an examination of several metabolites. Using Fisher’s exact test, scipy.stats (Python packages) (https://docs.scipy.org/doc/scipy/) was used to find statistically significantly enriched pathways.

### Targeted serum amino acid profile analysis

A total of 22 amino acids in bile samples were evaluated using the targeted UPLC-MS/MS method due to their significant differential abundance from the untargeted metabolomics analysis. For metabolite extraction, a nearly 50-μL bile sample was used, followed by 150 μL of acetonitrile, 30 min of sonication at 40 kHz, and 5 min of centrifugation at 13,000 rpm at 4°C. The supernatant was then added to the LC-MS/MS system for analysis.

At Majorbio Bio-Pharm Technology Co. Ltd. (Shanghai, China), the LC-MS/MS analysis of the sample was carried out using an ExionLC AD system coupled with a QTRAP® 6500+ mass spectrometer (AB Sciex, USA). Briefly, samples were separated by a Waters BEH Amide (100 * 2.1 mm, 1.7 μm) thermostated at 35°C. Separation of the metabolites was achieved at 1 mL/min flow rate with a mobile phase as a gradient that consisted of 95% acetonitrile in water with 0.4% formic acid and 20 mM ammonium formate (solvent A) and 95% acetonitrile in water with 0.4% formic acid and 20 mM ammonium formate (solvent B), 6 min of total chromatographic separation. The solvent gradient changed according to the following conditions: from 0% to 10% B, 0–1 min; from 10% to 15% B, 1–2.6 min; from 15% to 30% B, 2.6–3.5 min; hold at 30% B from 3.5 to 4.0 min; from 30% to 0% B, 4–4.1 min; hold at 0% B until the end of separation. During the period of analysis, all of these samples were stored at 4°C.

The mass spectrometric data were collected using a UHPLC coupled to a QTRAP® 6500+ mass spectrometer (AB Sciex, USA) equipped with an ESI source operating in positive mode. The source and gas settings were as follows: source temperature at 550°C; curtain gas (CUR) at 35 psi; CAD gas pressure medium; both ion source Gas1 and Gas2 at 70 psi; ISVF at 5,500 V in positive mode. Default parameters were used in AB Sciex quantitative software OS for automatic identification and integration of each ion fragment, and manual inspection was assisted.

### Statistical analysis

The results were expressed as means ± standard deviation (SD). SPSS Statistics software version 22 was used for statistical analysis, and GraphPad Prism version 8.0.1 was utilized to generate box plots and chart of columns (*P < 0.05).

## Results

### Clinical characteristics of the study subjects

Based on the demographic analysis, there were no statistically significant variations with age, alanine aminotransferase/aspartate aminotransferase (ALT/AST), and γ-glutamyltranspeptidase (GGT) between the OB (n = 25) and OB+C.s groups (n = 23) (*P* > 0.05). While in OB+C.s patients, total bilirubin (TBIL), direct bilirubin (DBIL), and indirect bilirubin (IBIL) were all significantly lower than those in OB patients (**Table 1**).

**Table 1:** Clinical characteristics of the study subjects.

	OB	OB+C.s	*t/χ2/z*	*P*
Number	25	23		
Female	15	10		
Age	60.04 ± 11.32	56.17 ± 9.49	1.276	ns
Laboratory data				
AST/ALT	0.7 (0.60, 1.15)	0.71 (0.48, 0.96)	-0.838	ns
GGT	405.00 (311.50, 518.50)	510.00 (220.00, 699.00)	-0.196	ns
TBIL	81.80 (32.40, 142.4)	25.20 (17.10, 72.10)	-3.313	*
DBIL	77.20 (22.90, 127.30)	9.50 (5.20, 42.70)	-4.056	*
IBIL	8.7 (5.95, 14.00)	17.90 (11.90, 25.70)	-2.910	*

Results are presented as medians (range, min–max)/mean ± standard deviation.

OB, biliary obstruction; OB+C.s, biliary obstruction with C. sinensis infection; AST, alanine aminotransferase; AST, aspartate aminotransferase; GGT, γ-glutamyltranspeptidase; ALP, alkaline phosphatase; TBIL, total bilirubin; DBI, direct bilirubin; IBIL, indirect bilirubin.

*Statistically significant results from Mann–Whitney U test.

The expression levels of inflammatory cytokines in the bile of OB and OB+C.s patients were analyzed (**Supplementary Figure S2**). The results showed that pro-inflammatory cytokines (IL-6 and TNF-α) and anti-inflammatory cytokine TGF-β displayed higher expression levels in the OB+C.s patients, while anti-inflammatory cytokine IL-10 had a higher expression level in the OB patients (*P* < 0.05).

### Bile metabolic profiles of OB and OB+C.s patients

**Figure 1:**
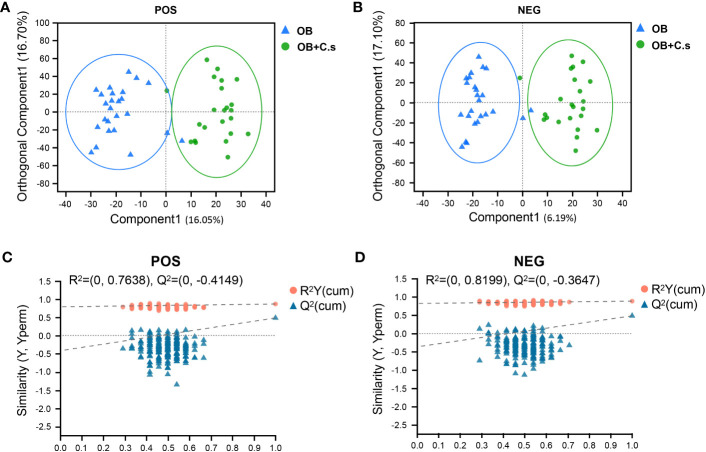
Multivariate analysis of LC-MS-based metabolomics data of OB and OB+C.s patients. **(A, B)** Orthogonal partial least squares discriminant analysis (OPLS-DA) in the positive (+) and negative (−) ion modes, respectively. **(C, D)** Permutation test results of the OPLS-DA mode in the positive (+) and negative (−) ion modes, respectively.

Unsupervised PCA score plots were first utilized to show the clustering behavior of bile metabolic profiles between OB and OB+C.s groups in order to depict the discrepancies between the two groups. The clearly separated trend, as seen in **Supplementary Figure S3**, demonstrated the presence of metabolic alterations. Then, OPLS-DA was conducted, which confirmed that there were considerable differences in the metabolic responses between the two groups in the ESI+ (**Figure 1A**) and ESI- (**Figure 1B**) modes. There was no overfitting in the OPLS-DA model according to the results of the permutation test (200 times) (**Figures 1C, D**).

### Differential metabolite analysis

**Figure 2:**
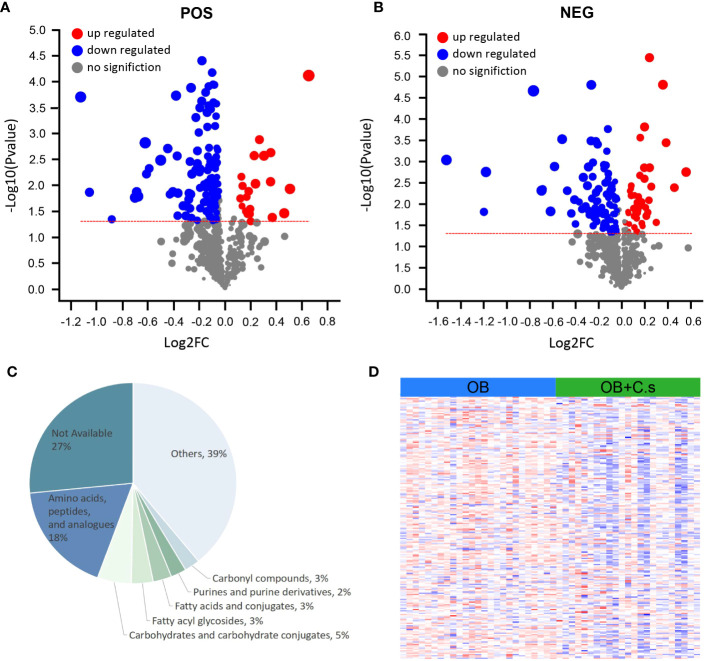
Differential Metabolite Analysis of OB+C.s versus OB patients. **(A, B)** Volcano plot representation of the differential metabolites identified in the ESI+ and ESI- ion modes, respectively. **(C)** Classification pie diagram of the differential metabolites at the subclass level. **(D)** Heatmap visualization of all differential bile metabolites.

Based on VIP > 1 and *P* < 0.05, a total of 146 and 132 significant metabolites contributing to class separation were selected in ESI+ and ESI- modes, respectively (**Figures 2A, B**; **Supplementary Table S1**). The results of different ionization modes (ESI+, ESI-) were combined. Finally, based on threshold values, the significant differential metabolites (n = 211, 98 from the ESI- mode and 113 from the ESI+ mode) were selected after removing non-endogenous, duplicate, and unknown metabolites (**Supplementary Table S2**). These metabolites mainly belonged to “Amino acids, peptides and analogues” and “Carbohydrates and carbohydrate conjugates” (**Figure 2C**). Among these 211 significant differential metabolites, 179 metabolites can be searched by HMDB or KEGG databases (**Supplementary Table S2**). In addition, the heat map was drawn to highlight the obvious difference of 211 metabolites in the two groups (**Figure 2D**).

### Metabolism pathways

**Figure 3:**
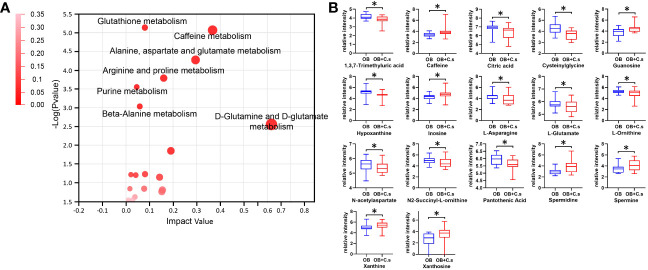
Metabolism pathway analysis of OB+C.s versus OB patients. **(A)** The size and color of each circle were based on pathway impact value and *P*-value, respectively. **(B)** The 17 key metabolites of OB+C.s versus OB patients in the ESI+ and ESI- ion modes. *P < 0.05.

**Figure 4:**
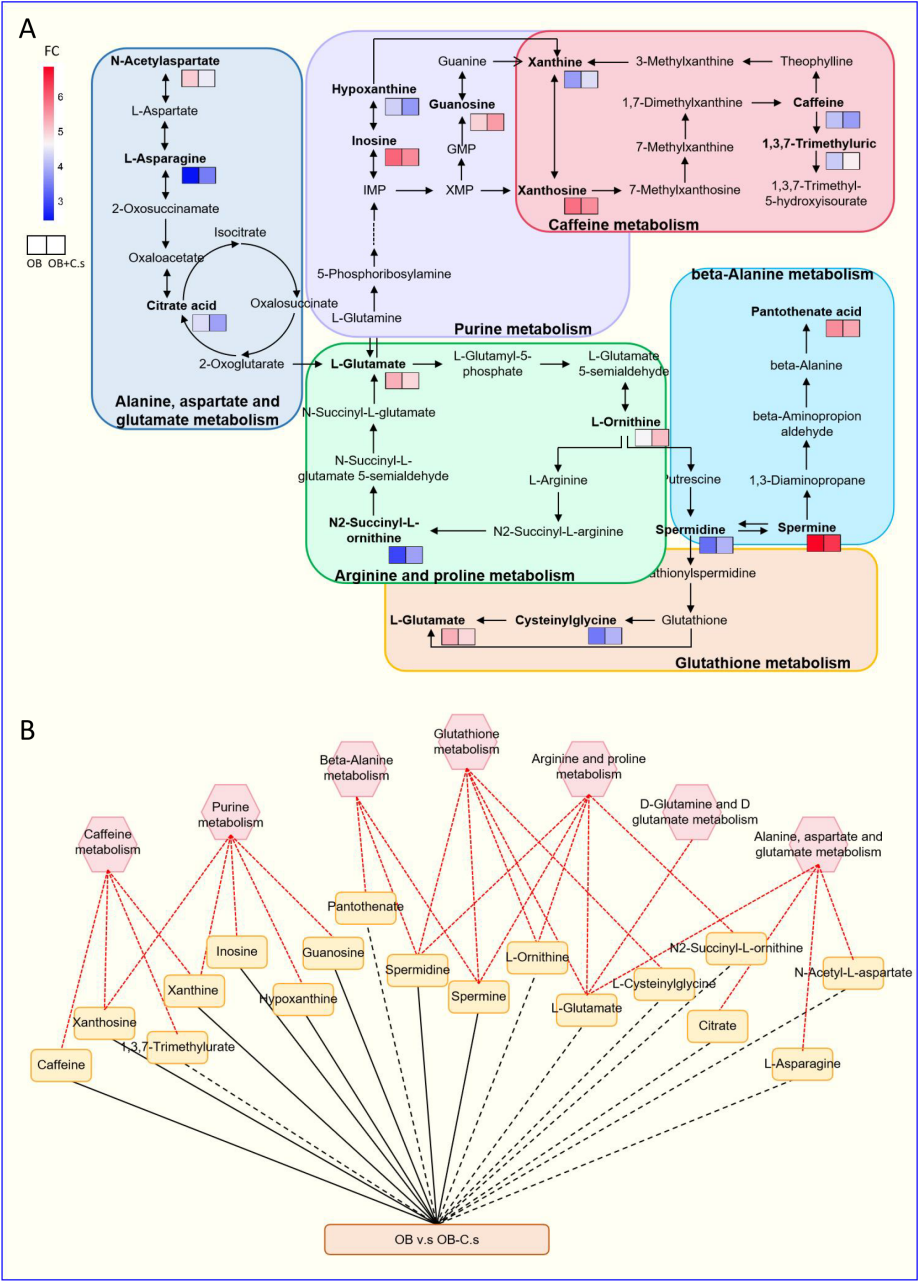
Integrated metabolic networks of significantly altered metabolic pathways of OB+C.s versus OB patients. **(A)** Metabolic map of OB+C.s versus OB patients. Log2(FC), an estimate of the log2-transformed ratio of the relative content of metabolites in OB+C.s patients to that in OB patients. **(B)** The 17 key metabolites of OB+C.s versus OB patients in the ESI+ and ESI- ion modes and their association with metabolic pathway diagram of the relationship between them. “Solid line” represents the increase of metabolites; “dotted line” represents the reduction of metabolites; “Red line” represents metabolite pathway.

Next, 211 discriminating metabolites were annotated into the KEGG database, and 35 metabolic pathways were obtained (**Figure 3A**; **Supplementary Table S3**). The metabolic pathways with impact value >0.02 and -log (*P* value) >2 were thought of as the most important pathways, including “Glutathione metabolism”, “Caffeine metabolism”, “Alanine, aspartate and glutamate metabolism”, “Arginine and proline metabolism”, “Purine metabolism”, “Beta-alanine metabolism”, and “D-glutamine and D-glutamate metabolism”. Compared with OB patients, a total of 17 key metabolites were screened, with eight metabolites upregulated and nine metabolites downregulated in OB+C.s patients (**Figure 3B**; **Supplementary Table S4**). As shown in **Figure 4A**, the most important pathways were integrated analyzed in the network. Moreover, **Figure 4B** showed the key metabolite changes and their association with the metabolic pathway diagram of the relationship between them in OB+C.s patients compared with OB patients. For example, Spermidine and Spermine were involved in “Beta-alanine metabolism”, “Glutathione metabolism”, and “Arginine and proline metabolism”, while L-glutamate participates in “Glutathione metabolism”, “Arginine and proline metabolism”, and “Alanine, aspartate and glutamate metabolism”.

### Differences in amino acid profiles between OB and OB+C.s patients analyzed by targeted metabolomics

**Figure 5:**
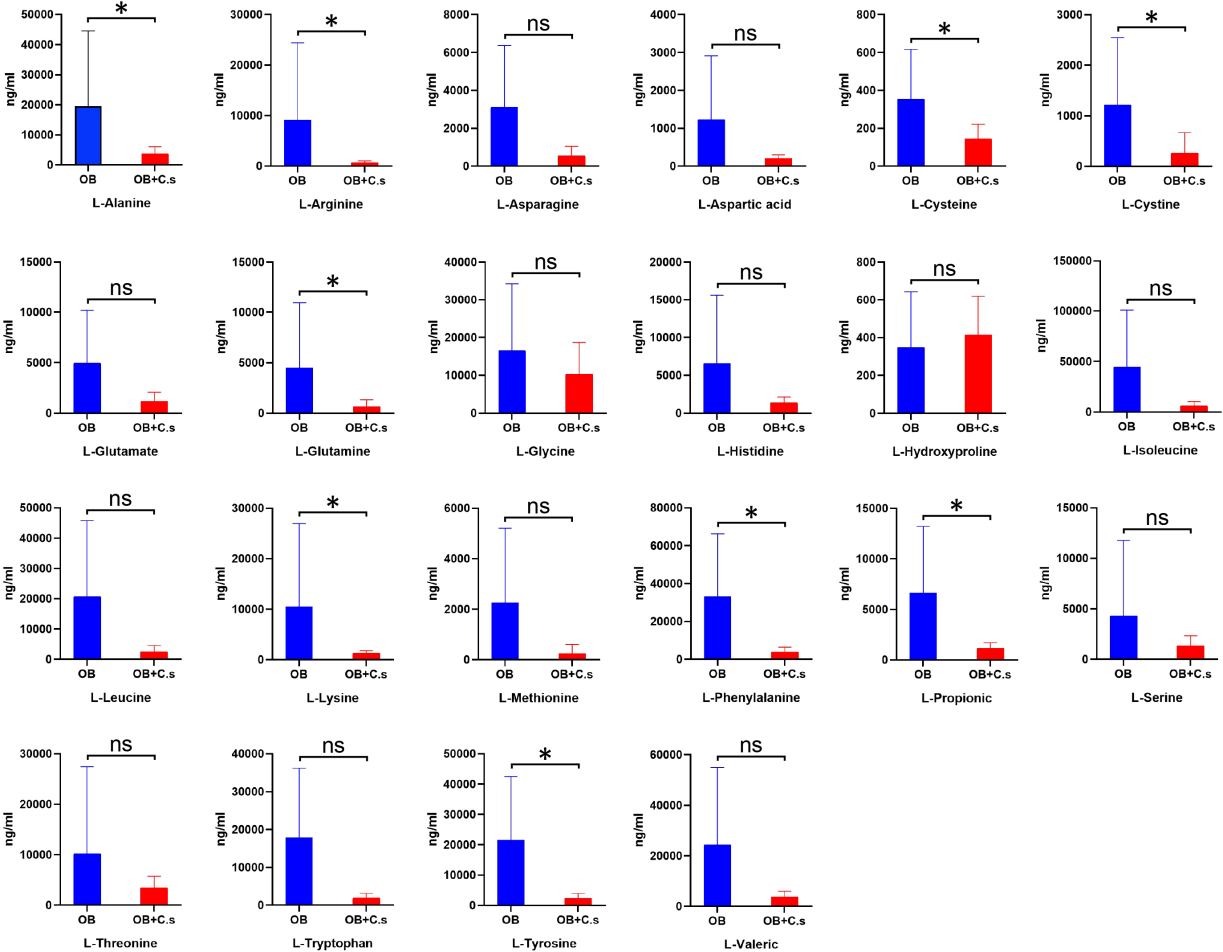
The concentrations of 22 amino acids in bile of OB+C.s versus OB patients. *P < 0.05, t-test. ns, no significant change.

According to untargeted metabolomics analysis, we suggested that the amino acid profile may have the ability to distinguish OB+C.s patients from OB patients. Therefore, targeted UPLC-MS/MS was used to further quantitatively examine 22 amino acids in bile samples (**Figure 5**). Among them, nine amino acids were significantly decreased in OB+C.s, including L-alanine, L-arginine, L-cysteine, L-cystine, L-glutamine, L-lysine, L-propionic, L-tryptophan, and L-tyrosine.

## Discussion

In this study, we identified differential metabolites and metabolic pathways between OB+C.s patients and OB patients. Untargeted metabolomics analysis of bile samples using GC/MS detected 211 metabolites. Multivariate analyses demonstrated significant separation between OB+C.s patients and OB patients in ESI+ mode and ESI− mode, respectively. KEGG analysis revealed that seven most important metabolic pathways were markedly altered between OB+C.s patients and OB patients. Most of these pathways were involved in amino acid metabolism, indicating that amino acid metabolism may be very important in the *C. sinensis* infection. Therefore, it is critical to comprehend the metabolic difference between OB+C.s and OB patients.

Amino acid, as the basic substance of protein, is one of the essential nutrients of organisms, which is widely involved in a variety of physiological activities (Luscombe-Marsh et al., 2016; Al-Koussa et al., 2020). Several studies have suggested that the levels and metabolism of amino acids are intimately associated with parasitic growth and the development of diseases (Muxel et al., 2019; Li et al., 2020). In our earlier research, the alteration of amino acid metabolism was observed in rat spleen and serum infected with *C. sinensis* (Zhang et al., 2020; Han et al., 2023). In addition, Qiu et al. (2022) found that the amino acid levels in the serum of rabbits were altered after *C. sinensis* infection. Similarly, the alteration of amino acid metabolism was also observed in *Schistosoma japonicum*-infected mouse serum (Huang et al., 2020). We also observed a significant difference in amino acid levels in OB+C.s patients compared to OB patients. As such, we suggested that amino acids may be important metabolites in distinguishing OB+C.s patients from OB patients.

Untargeted metabolomics analysis showed lower levels of glutamate (*P* < 0.05) in the bile of OB+C.s patients, which was involved in “Glutathione metabolism”, “Arginine and proline metabolism”, “Alanine, aspartate and glutamate metabolism”, and “D-glutamine and D-glutamate metabolism”. And targeted metabolomics analysis showed lower levels of glutamate (*P* > 0.05) and glutamine (*P* < 0.05) in the bile of OB+C.s patients. As an immediate precursor for the synthesis of glutathione, glutamate is a crucial element of defense against oxidative stress (Amores-Sanchez and Medina, 1999; Johnson et al., 2003). Except that, it has been considered an important immunomodulatory, and immune system cells express a number of glutamate receptors (Pacheco et al., 2007). Glutamate is a highly abundant non-essential amino acid that is produced from glutamate by the enzyme glutamine synthetase. It is involved in cell growth and proliferation, the production of other non-essential amino acids, antioxidative defense, and other processes (Tan et al., 2017; Wappler et al., 2020). Additionally, a decrease in the quantity of extracellular glutamine makes cells more susceptible to apoptosis (Chen and Cui, 2015). Studies have shown that the level of glutamine was significantly reduced in the serum of rabbits infected with *C. sinensis* (Qiu et al., 2022). Therefore, we speculated that glutamate and glutamine may play important roles in distinguishing OB+C.s patients from OB patients.

In addition, OB+C.s patients showed a lower level of bile arginine in untargeted metabolomics analysis (*P* > 0.05, **Supplementary Table S1**) and targeted metabolomics analysis (*P* < 0.05), which participates in “Arginine and proline metabolism”. L-arginine is a basic semi-essential amino acid. A considerable amount of L-arginase is released into the blood to accelerate the consumption of arginine, which also results in a lack of arginine, when the animal body is damaged (particularly the liver) or stressed (Zou et al., 2019; Marti and Reith, 2021). Previous research has shown that arginine metabolic changes were connected to acute liver damage in rats (Saitoh et al., 2014). By generating NO, arginine, a precursor of citrulline, controls the immune system function in the majority of cell types (Rath et al., 2014). In mammals, arginine can also be catalyzed to polyamines like putrescine, spermidine, and spermine (Morris, 2016). Untargeted metabolomics analysis showed higher levels of spermidine and spermine (*P* < 0.05) in the bile of OB+C.s patients, which are involved in “Beta-alanine metabolism”, “Glutathione metabolism”, and “Arginine and proline metabolism”. Spermine and spermidine, as Reactive Oxygen Species (ROS) scavengers, can protect DNA from free radical attacks, regulating cell proliferation, differentiation, and apoptosis (Pegg, 2009; Wang et al., 2020). We considered that *C. sinensis* infection caused a disturbance of the arginine metabolic pathway in the bile of patients with biliary obstruction, which is important to distinguish OB+C.s patients from OB patients.

To limit clinical heterogeneity, we applied strict inclusion criteria in our study. However, due to the great difficulty in collecting bile samples from healthy people, we did not include a normal control group in our study. Furthermore, due to the small sample size, we did not divide the patients into groups according to sex or age, two factors that may affect how different metabolomic profiles appear. Thus, alternative models including murine, cell cultures, and organoids would be necessary for future experimental methods.

## Conclusion

In conclusion, based on LC-MS/MS-based metabolomics, we found that differential bile metabolites, including amino acids (asparagine, glutamate, glutamine, and arginine), changed significantly in OB+C.s compared to OB patients. Some altered metabolic pathways, such as “Glutathione metabolism”, “Arginine and proline metabolism”, “Alanine, aspartate and glutamate metabolism”, “D-Glutamine and D-glutamate metabolism”, and “Beta-Alanine metabolism”, may provide an important basis for distinguishing the OB+C.s patients from OB patients.

## Data availability statement

The datasets presented in this study can be found in online repositories. The names of the repository/repositories and accession number(s) can be found below: MetaboLights: MTBLS8376.

## Ethics statement

The studies involving humans were approved by The Ethics Committee of Harbin Medical University. The studies were conducted in accordance with the local legislation and institutional requirements. The participants provided their written informed consent to participate in this study.

## Author contributions

Xu-LZ and Xi-LZ conceived and designed the experiments. Xu-LZ, Xi-LZ and SH performed the experiments, drafted the first manuscript and participated in trial communication and monitoring. XJ, SD, YG, BS, JD, XL, BS, XH and WZ modified the manuscript the carried out statistical calculations. All authors participated in revision of the manuscript and approved the final version.

## Supplementary material

Supplementary Figure 1
*C. sinensis* eggs in bile of OB+C.s patients.

Supplementary Figure 2 Analysis of inflammatory cytokines in the bile of OB+C.s and OB patients.

Supplementary Figure 3 Principal component analysis (PCA) score scatter plots of OB+C.s patients versus OB patients. **(A)** PCA score scatter plot of splenic metabolites in the positive ion mode (ESI+). **(B)** PCA score scatter plot of splenic metabolites in the negative ion mode (ESI−). OB+C.s, biliary obstruction; OB+C.s biliary obstruction with *C. sinensis* infection; QC, quality control.
