# Supplementary figures and images for: Comparative analysis of bile metabolic profile in patients with biliary obstruction complicated by Clonorchis sinensis infection
Source: Front Cell Infect Microbiol. 2023 Sep 12;13:1254016. doi: 10.3389/fcimb.2023.1254016 (PMC10585366; doi:10.3389/fcimb.2023.1254016)

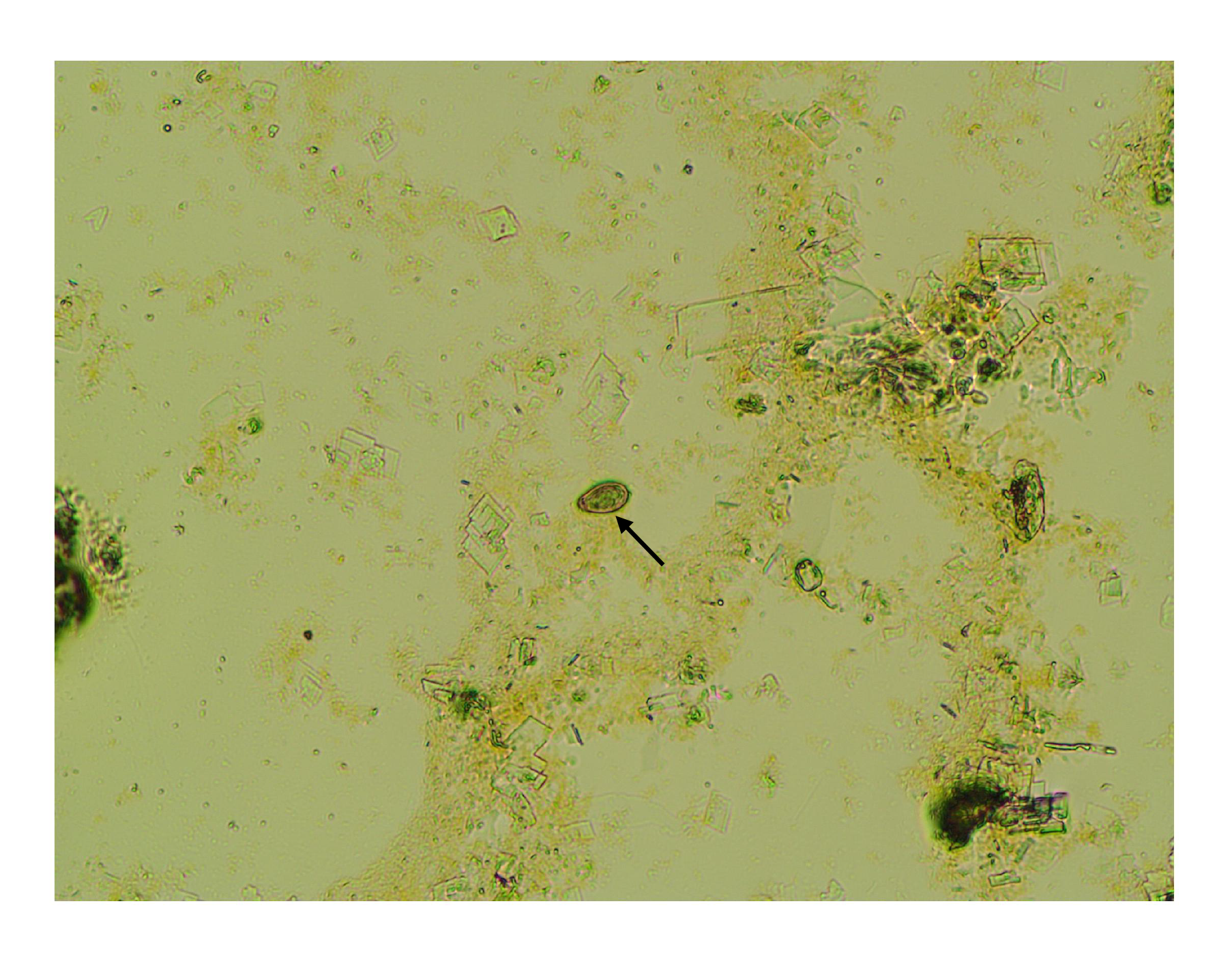

Supplement: Supplementary Figure 1 — C. sinensis eggs in bile of OB+C.s patients. [file Image_1.tif]

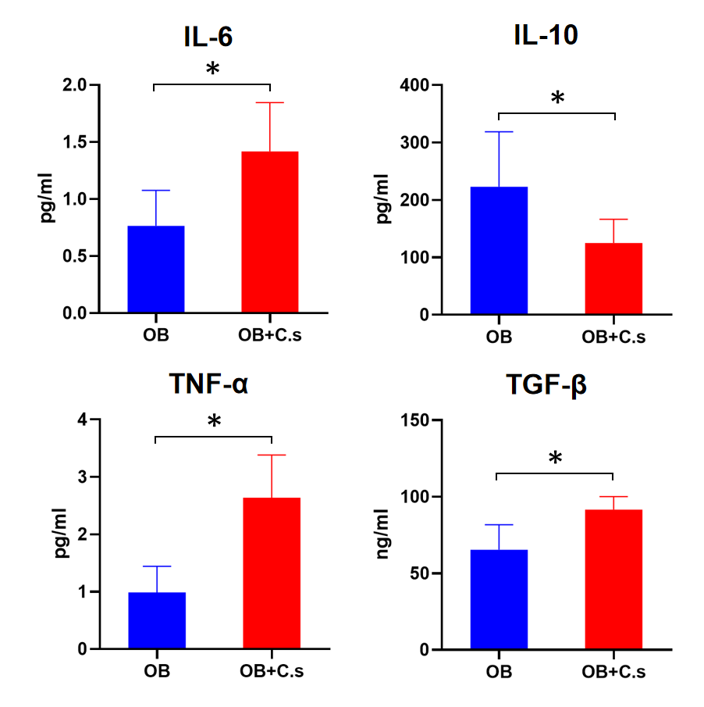

Supplement: Supplementary Figure 2 — Analysis of inflammatory cytokines in the bile of OB+C.s and OB patients. [file Image_2.tif]

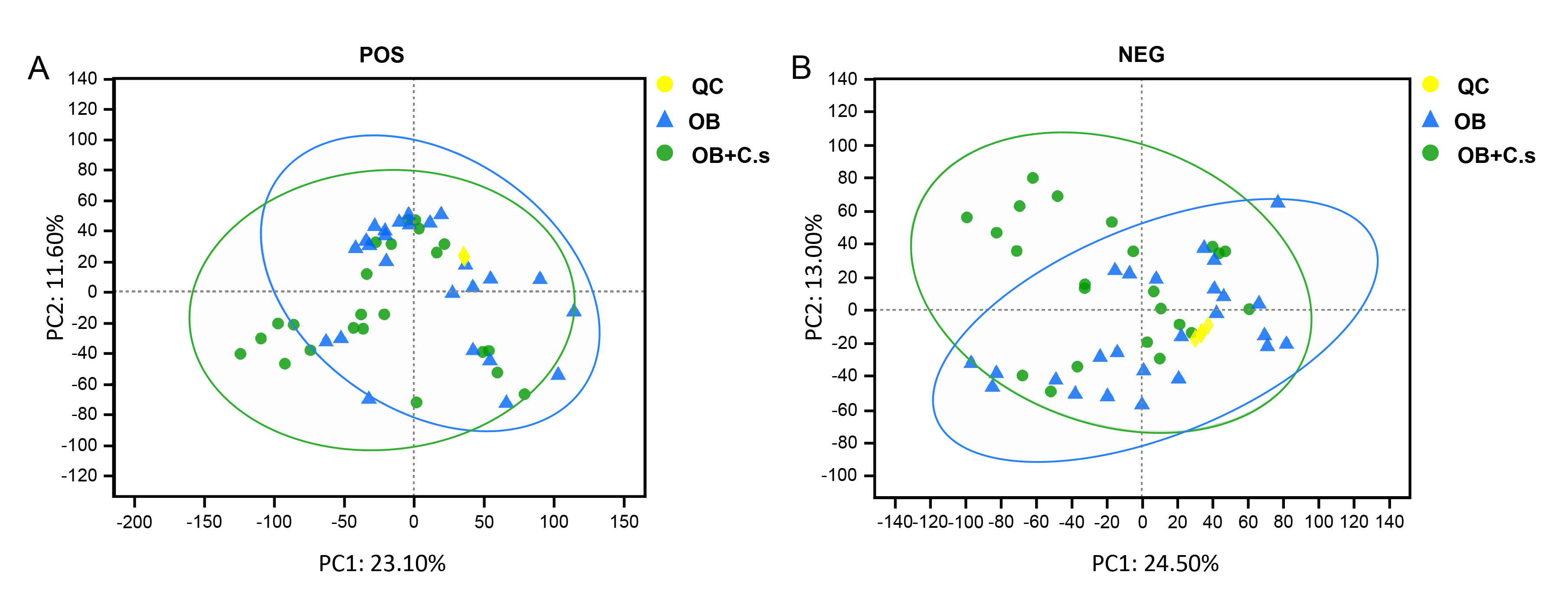

Supplement: Supplementary Figure 3 — Principal component analysis (PCA) score scatter plots of OB+C.s patients versus OB patients. (A) PCA score scatter plot of splenic metabolites in the positive ion mode (ESI+). (B) PCA score scatter plot of splenic metabolites in the negative ion mode (ESI−). OB+C.s, biliary obstruction; OB+C.s biliary obstruction with C. sinensis infection; QC, quality control. [file Image_3.tif]
